# The Cross-Talk between Mesenchymal Stem Cells and Immune Cells in Tissue Repair and Regeneration

**DOI:** 10.3390/ijms22052472

**Published:** 2021-03-01

**Authors:** Carl Randall Harrell, Valentin Djonov, Vladislav Volarevic

**Affiliations:** 1 Regenerative Processing Plant, LLC, 34176 US Highway 19 N Palm Harbor, Palm Harbor, FL 34684, USA; dr.harrell@regenerativeplant.org; 2 Institute of Anatomy, University of Bern, Baltzerstrasse 2, 3012 Bern, Switzerland; valentin.djonov@ana.unibe.ch; 3 Center for Molecular Medicine and Stem Cell Research, Department of Microbiology and Immunology, Faculty of Medical Sciences, University of Kragujevac, 69 Svetozar Markovic Street, 34000 Kragujevac, Serbia

**Keywords:** mesenchymal stem cells, immune cells, immunomodulation, angiogenesis, regeneration

## Abstract

Mesenchymal stem cells (MSCs) are self-renewable, rapidly proliferating, multipotent stem cells which reside in almost all post-natal tissues. MSCs possess potent immunoregulatory properties and, in juxtacrine and paracrine manner, modulate phenotype and function of all immune cells that participate in tissue repair and regeneration. Additionally, MSCs produce various pro-angiogenic factors and promote neo-vascularization in healing tissues, contributing to their enhanced repair and regeneration. In this review article, we summarized current knowledge about molecular mechanisms that regulate the crosstalk between MSCs and immune cells in tissue repair and regeneration.

## 1. Introduction

Tissue regeneration is a result of a healing process which completely restores the architecture and function of damaged tissue [1]. Some tissues are more capable of cellular proliferation and regeneration than others [2]. Epithelia and hematopoietic tissues, so-called continuously dividing tissues, contain rapidly proliferating stem cells which constantly proliferate in order to replace dead or sloughed-off cells [1,2]. Quiescent or stable tissues, including liver, kidney and pancreatic tissues, are composed of cells that normally exist in a non-dividing state but may enter the cell cycle in response to certain stimuli, such as cell injury [2]. In contrast to stable tissues, permanent tissues (cardiac and skeletal muscles) are composed of cells that have left the cell cycle permanently and are therefore unable to proliferate [2]. The severe damage of quiescent tissues and the injury of permanent tissues are usually repaired by the laying down of connective tissue (a process commonly referred to as scarring), which results in the structural and functional abnormalities of the affected organ [1,2].

The immune system regulates tissue healing and regeneration [1,2,3]. Danger-associated molecular patterns (DAMPs) and alarmins, released from damaged cells, attract circulating leucocytes to the site of injury [3]. Accordingly, circulating neutrophils, monocytes, dendritic cells (DCs), natural killer (NK) cells, innate lymphoid cells (ILC) and T lymphocytes, are mobilized by injured cells [1]. Immediately after injury, innate immune cells (pro-inflammatory DCs, N1 neutrophils, M1 macrophages, NK1 and NK17 cells) release various inflammatory cytokines (tumor necrosis factor alpha (TNF-α), interleukin (IL)-1β, IL-6, IL-8) and chemokines, which trigger an acute phase of inflammatory response [1,2,3]. After the successful elimination of pathogens, immune cells acquire an immunosuppressive phenotype [3]. Anti-inflammatory N2 neutrophils, M2 macrophages and tolerogenic DCs produce anti-inflammatory cytokines, trophic and growth factors that promote the generation and expansion of immunosuppressive cells (NK regulatory cells (NKregs) and T regulatory cells (Tregs)) which co-operate with immunosuppressive cells of innate immunity to attenuate on-going inflammation [1,2,3]. Since sustained inflammation impairs the healing process, its resolution is necessary for the restoration of tissue homeostasis [1,2,3]. Therefore, therapeutic agents which are used in regenerative medicine should efficiently inhibit detrimental immune response, promote the expansion of immunosuppressive immune cells and prevent the inflammation-induced death of parenchymal cells [4].

Mesenchymal stem cells (MSCs) are self-renewable, rapidly proliferating, multipotent stem cells which reside in almost all post-natal tissues, including quiescent and permanent tissues [5]. MSCs spontaneously differentiate into the cells of mesodermal origin (adipocytes, chondrocytes, and osteocytes) and, under specific conditions, are capable of generating cells of neuro-ectodermal (neurons, astrocytes, and oligodendrocytes) and endodermal origin (hepatocytes) [5]. In addition to their great differentiation potential, MSCs possess potent immunomodulatory and angiomodulatory characteristics [6]. In juxtracrine and paracrine manners, MSCs regulate the phenotype and function of all immune cells that participate in tissue repair and regeneration [5,6]. Under the influence of alarmins and inflammatory cytokines, released from injured cells, MSCs generate anti-inflammatory phenotype, induce the generation and expansion of immunosuppressive M2 macrophages, tolerogenic DCs, NKregs and Tregs, efficiently alleviating detrimental immune response and on-going inflammation [6]. By producing a large number of immunomodulatory molecules (transforming growth factor-β (TGF-β), hepatic growth factor (HGF), nitric oxide (NO), indolamine 2,3-dioxygenase (IDO), interleukin (IL)-10, IL-6, IL-1 receptor antagonist (IL-1Ra), hemeoxygenase-1 (HO-1), prostaglandin E2 (PGE2), TNF-α stimulated gene/protein 6 (TSG-6)) and pro-angiogenic factors (vascular endothelial growth factor (VEGF), angiopoietin-1, placental growth factor (PGF), HGF, basic fibroblast growth factor (bFGF), TGF-β, platelet-derived growth factor (PDGF), IL-6), MSCs regulate immune response and vasculogenesis, crucially contributing to the enhanced repair of injured tissues [7]. In this review article, we summarized current knowledge about molecular mechanisms that regulate the cross-talk between MSCs and immune cells in the process of tissue regeneration. An extensive literature review was carried out in January 2021 across several databases (MEDLINE, EMBASE, Google Scholar), from 1990 to present. Keywords used in the selection were: “mesenchymal stem cells”, “immune cells”, “neutrophils”, “monocytes”, “macrophages”, “dendritic cells”, “natural killer cells”, “innate lymphoid cells”, “T cells” “inflammation”, “tissue repair”, “regeneration”, “immunosuppression”. Studies that emphasized molecular and cellular mechanisms responsible for MSC-dependent immunomodulatory effects in the healing of injured tissues were analyzed in this review.

## 2. The Crosstalk between MSCs and Neutrophils in Tissue Repair and Regeneration

Neutrophils, in time-dependent manner, contribute to tissue repair via multiple mechanisms [8]. Within minutes after tissue damage, neutrophils migrate at the site of injury and, as professional phagocytes, clear necrotic tissue and cellular debris by phagocytosis [9]. After the elimination of microbial pathogens and cellular debris, neutrophils participate in the restoration of tissue homeostasis [9]. By removing cellular remnants, neutrophils prevent DAMPs-driven recruitment of inflammatory cells in healing tissues [8,9]. Additionally, neutrophils release neutrophil extracellular traps (NETs) that capture monocyte and lymphocyte-attracting chemokines and express chemokine receptors that can function as scavengers to reduce the availability of pro-inflammatory chemokines for the recruitment of additional circulating leucocytes [9]. Moreover, neutrophils produce matrix metalloproteinase (MMP)-9 which is capable of degrading DAMPs, particularly HMGB1 and HSP90, further dampening the recruitment of leucocytes into the site of injury [8,9,10].

A sub-population of CXCR4+VEGFR+CD49d+ neutrophils, which is abundantly present in the injured tissues, release large amounts of pro-angiogenic factors, including VEGF, TGF-β, IL-6, which stimulate neo-angiogenesis and promote tissue repair [8,11]. These pro-angiogenic neutrophils generate new blood vessels and enable the better delivery of oxygen, growth and trophic factors in ischemic regions, facilitating tissue regrowth and regeneration [10,11].

During the healing phase of tissue repair, the majority of neutrophils acquire the immunosuppressive N2 phenotype [1,3,8]. Anti-inflammatory N2 neutrophils produce immunosuppressive cytokines (IL-10 and TGF-β) and release microvesicles containing annexin A1 which induce macrophage phenotype switching toward an immunosuppressive and “pro-repair” M2 phenotype [8,9]. Additionally, after the removal of cellular remnants, neutrophils undergo apoptosis, expose phosphatidyl-serine on their surface and become phagocyted by resident macrophages [9]. The phagocytosis of apoptotic neutrophils further induces macrophage phenotype switching towards anti-inflammatory M2 phenotype [3,8]. M2 macrophages, in turn, release various pro-resolving mediators, crucially contributing to the enhanced repair of injured tissue [8,9].

**Figure 1:**
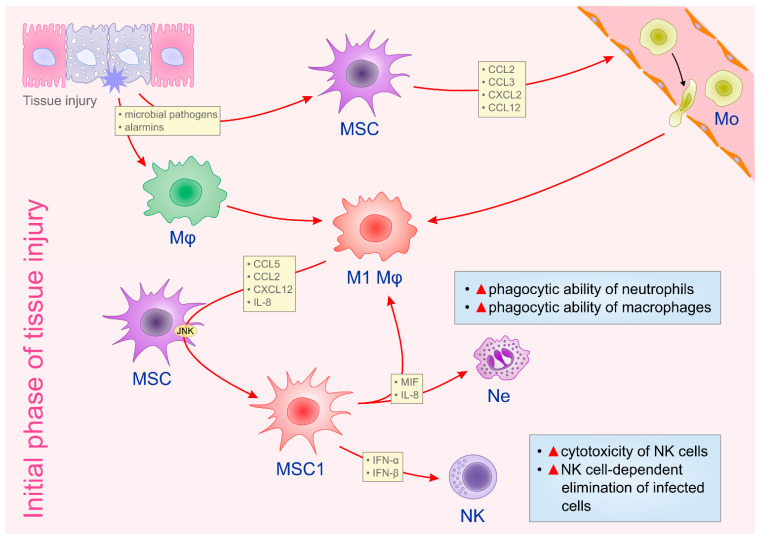
The crosstalk between MSCs and immune cells in the initial phase of tissue injury. Immediately upon tissue injury, resident MSCs become activated by microbial pathogens and/or by alarmins released from injured cells. Activated MSCs produce monocyte-attracting chemokines (CCL2, CCL3, CXCL2, CCL12) which recruit circulating monocytes to the site of injury and inflammation. Tissue resident macrophages and recruited monocytes obtain pro-inflammatory (M1) phenotype upon phagocytosis of microbial pathogens and apoptotic cells. M1 macrophages/monocytes produce CCL5, CCL2, CXCL12, IL-8 which activate JNK-dependent signaling pathway in resident MSCs, inducing their conversion in inflammatory (MSC1) cells. MSC1 secrete anti-viral cytokines (IFN-α and IFN-β) that up-regulate cytotoxicity of NK cells and enhance NK cell-dependent elimination of infected cells. Additionally, MSC1, in an IL-8- and MIF-dependent manner, enhance the phagocytic ability of neutrophils and macrophages, enabling the efficient removal of apoptotic cells, necrotic tissue and cellular debris. Abbreviations: mesenchymal stem cells (MSCs), C–C motif chemokine ligand (CCL), C-X-C motif chemokine ligand (CXC), c-JunNH2-terminal kinase (JNK), macrophage migration inhibitory factor (MIF), natural killer (NK) cells, interleukin-8 (IL-8), interferon alpha (IFN-α), interferon beta (IFN-β).

**Figure 2:**
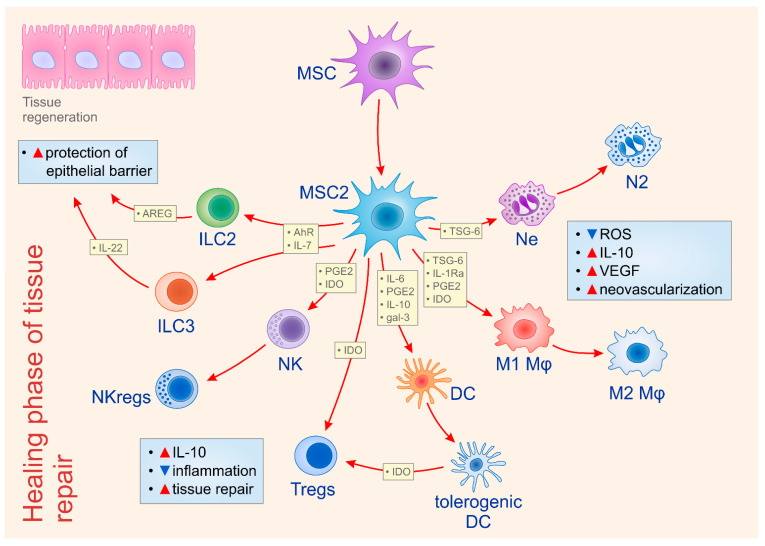
Molecular mechanisms responsible for MSC-dependent modulation of immune cells during the healing phase of tissue repair and regeneration. During the resolution phase of tissue repair, MSCs obtain anti-inflammatory and immunosuppressive (MSC2) phenotype. MSC2 secrete large amounts of immunosuppressive and angio-modulatory factors that promote tissue repair by alleviating ongoing inflammation and by promoting neo-vascularization in regenerated tissues. MSC2, in a juxtracrine manner (through the activation AhR) and in paracrine manner (through the secretion of IL-7) induce proliferation and activation of AREG-expressing ILC2 and IL-22-producing ILC3 which protect the integrity of epithelial cell barrier. MSC2 produce TSG-6 which reduces production of reactive oxygen species (ROS) and induces enhanced expression of IL-10 in neutrophils, favoring their polarization in immunosuppressive (N2) phenotype. MSCs, in TSG-6, IL-1Ra, PGE2 and IDO-dependent manner, induce conversion of TNF-α and IL-1β producing inflammatory (M1) macrophages into immunosuppressive, IL-10 producing (M2) cells. MSC-derived immunomodulatory factors (IL-6, PGE2, IL-10 and galectin-3) induce the generation of the tolerogenic phenotype in DCs. MSC2 and tolerogenic DCs, in an IDO-dependent manner, induce the generation and expansion of immunosuppressive Tregs. Similarly, MSC2, in a PGE2 and IDO-dependent manner, induce the polarization of inflammatory NK cells into IL-10-producing, anti-inflammatory NKregs. Tregs and NKregs produce immunosuppressive cytokines that attenuate on-going inflammation and enable more efficient tissue repair. Additionally, MSC2 induces increased production of VEGF in N2 neutrophils and M2 macrophages, contributing to the better neovascularization of healing tissues. Abbreviations: aryl hydrocarbon receptor (AhR), reactive oxygen species (ROS), interleukin (IL), Amphiregulin (AREG), innate lymphoid cells (ILC), interleukin 1 receptor antagonist (IL-1Ra), prostaglandin E2 (PGE2), TNF-α stimulated gene/protein 6 (TSG-6), indolamine 2,3-dioxygenase (IDO), T regulatory cells (Tregs), NK regulatory cells (NKregs), vascular endothelial growth factor (VEGF).

MSCs modulate phenotype and function of neutrophils in all phases of tissue repair and regeneration [12]. During an initial phase of tissue healing, resident MSCs, in IL-8 and macrophage migration inhibitory factor (MIF)-dependent manner, enhance the phagocytic ability of neutrophils, contributing to the efficient removal of necrotic tissue and cellular debris (Figure 1) [13]. Additionally, MSCs, via up-regulation of the extracellular superoxide dismutase (SOD3), prevent neutrophil death and enhance neutrophil-dependent elimination of microbial pathogens and cellular remnants [13]. On the contrary, during the resolution phase of tissue repair, MSCs reduce the presence of pro-inflammatory N1 neutrophils and induce their conversion in anti-inflammatory N2 cells (Figure 2) [12,13]. Intercellular adhesion molecule 1 (ICAM-1)-dependent engulfment of neutrophils is mainly responsible for MSC-based elimination of N1 neutrophils [13]. Additionally, MSCs produce TSG-6, which reduces the production of reactive oxygen species (ROS) and induces enhanced expression of IL-10 in neutrophils, favoring their polarization in immunosuppressive N2 phenotype [14]. The inhibition of extracellular signal regulated kinase (ERK) pathway seems crucially responsible for the MSC-dependent generation of N2 phenotype in neutrophils [15]. By using dextran sodium sulphate (DSS)-induced colitis, a murine model of ulcerative colitis in which N1 neutrophils play important role in disease progression and N2 neutrophils in disease regression, Wang and colleagues demonstrated that MSCs, by modulating ERK phosphorylation, induced polarization of N1 neutrophils in N2 immunosuppressive cells, resulting in enhanced mucosal healing of DSS-injured colons [15]. Significantly reduced numbers of ICAM-1, FAS, and CCL3-expressing N1 neutrophils and increased numbers of CCL2, CXCR4-expressing N2 neutrophils were observed in colon tissue samples of DSS-treated mice that received MSCs [15]. Additionally, MSCs induce an increased production of VEGF in neutrophils, contributing to the better neovascularization of healing tissues [11]. Similarly, pro-angiogenic neutrophils promote the expression of PDGF, angiopoietin-1, IL-6 and HGF in MSCs, enhancing their pro-angiogenic properties [11,12]. Accordingly, crosstalk between pro-angiogenic neutrophils and MSCs results in the enhanced proliferation of endothelial cells and vascular regeneration in healing tissues [11,12].

## 3. An interplay between MSCs and Macrophages in Tissue Repair and Regeneration

Macrophages are critically involved in normal tissue homeostasis and exhibit an important regulatory activity at all stages of repair and regeneration of damaged tissues [16]. Immediately after injury, tissue resident macrophages act as scavenger cells which phagocyte cellular debris, pathogens, apoptotic neutrophils, and dying cells [16]. DAMPs and pathogen associated molecular patterns (PAMPs) activate toll-like receptors (TLRs) in macrophages which acquire pro-inflammatory M1 phenotype and orchestrate the initial cellular response following injury [17]. M1 macrophages secrete CCL2, MMP-12, nitric oxide (NO), inflammatory cytokines (TNF-α, IL-1β, IL-12) and various other inflammatory chemokines, enabling the increased recruitment of circulating leucocytes to the site of injury [17]. In the initial phase of tissue healing, macrophages act as phagocytes and clear apoptotic cells and cellular debris [17]. After the early inflammatory phase subsides, the predominant macrophage population assumes a wound healing M2 phenotype characterized by the low expression of Ly6C and CCR2 and high expression of CX3CR1, CD206 and CD163 [16,17]. M2 macrophages, through the production of numerous growth factors (PDGF, TGF-β1, insulin growth factor (IGF)-1, and VEGF-α), promote cellular proliferation, neo-angiogenesis and, in the case of severe injury, activation and differentiation of tissue resident stem and progenitor cells [16]. M2 macrophages also produce soluble mediators (IL-13, TGF-β1) that induce the differentiation of fibroblasts into myofibroblasts which, through the increased synthesis of extracellular matrix (ECM) components, enable wound contraction and closure [18]. In order to prevent the excessive deposition of collagen and ECM proteins, at the final phase of tissue healing, the majority of the M2 macrophages obtain anti-inflammatory phenotype, characterized by increased capacity for the production of immunosuppressive cytokine IL-10 [16,17]. Additionally, anti-inflammatory M2 macrophages secrete ECM-degrading MMPs (MMP-2, MMP-9, and MMP-13) and prevent fibrosis [18]. These anti-inflammatory macrophages express program death ligand (PD-L)1 and PD-L2, that play major roles in suppressing other pro-inflammatory and pro-fibrotic immune cells, enabling alleviation of on-going inflammation and fibrosis [16,17,18].

Since M1 and M2 macrophages play critical roles at different stages of tissue repair and regeneration, MSCs support the phagocytic properties of M1 macrophages in the initial phase of tissue healing, while, at the final stage of tissue repair, promote the generation and expansion of anti-inflammatory M2 macrophages [19].

At the initial stage of tissue injury, microbial invasion activates tissue resident MSCs [6]. After sensing pathogens in inflamed tissues, MSCs produce monocyte-attracting chemokines (CCL2, CCL3, CXCL2, CCL12), which promote the egression of monocytes from the bone marrow and enable their recruitment into the site of injury and inflammation [6,19,20]. After the phagocytosis of microbial pathogens and apoptotic cells, macrophages obtain pro-inflammatory M1 phenotype and produce MSC-attracting chemokines and cytokines CCL5, CCL2, CXCL12 and IL-8 [19,20]. These inflammatory mediators activate c-JunNH2-terminal kinase (JNK)-dependent signaling pathway in MSCs and induce their conversion in inflammatory, IFN-γ- and TNF-α-producing MSC1 cells, which, together with N1 neutrophils and M1 macrophages, participate in the elimination of microbial pathogens and cellular debris (Figure 1) [6,19].

During the healing phase of tissue repair, MSCs, in TSG-6, PGE2 and IDO-dependent manner, induce conversion of TNF-α and IL-1β producing inflammatory M1 macrophages into immunosuppressive, IL-10 producing M2 cells that attenuate on-going inflammation and promote tissue regeneration [6,7]. MSC-derived TSG-6 interact with CD44 on macrophages to decrease TLR2/NFκ-B signaling and consequently alleviate the secretion of inflammatory mediators, particularly NO, TNF-α and IL-1β [21,22]. M1 macrophage-sourced IL-1β, is considered as an important regulator of persistent inflammation and fibrosis [18]. Importantly, IL-1β, released from activated macrophages, induces the generation of the immunosuppressive phenotype in tissue resident MSCs [6,18,19]. IL-1β-primed MSCs increase the production of anti-inflammatory cytokines, IL-10 and IL-1Ra [18,19]. MSC-derived IL-1Ra, a naturally occurring inhibitor of IL-1β, has crucially important role in MSC-based suppression of M1 macrophages-driven inflammation [23]. When IL-1Ra binds to the IL-1 receptor (IL-1R), the interaction between inflammatory IL-1 and IL-1R is prevented [23]. The apoptosis of parenchymal cells, synthesis and release of matrix-degrading enzymes and chemokines, as well as other inflammatory events, initiated by IL-1:IL-1R interaction, are inhibited by MSC-sourced IL-1Ra [22,23].

In addition to TSG-6 and IL-1Ra, MSC-sourced IL-6 and PGE2 also showed the ability to transform inflammatory, TNF-α and IL-1β-producing M1 macrophages into IL-10-secreting, anti-inflammatory M2 cells (Figure 2) [21,22]. MSC-derived IL-6 and PGE2 binds to IL-6R and EP2 and EP4 receptors on macrophages and promotes production of immunosuppressive IL-10, which, in turn, in autocrine and paracrine manners, favors the generation of M2 macrophages which participate in tissue repair and regeneration [21,22].

The interplay between M2 macrophages and MSCs plays an essential role in the vascular regeneration of injured tissues [19]. Since MSCs represent a valuable source of pro-angiogenic VEGF and angiopoentin-1, the transplantation of autologous MSCs efficiently repaired corneal wounds by promoting local tissue neo-vascularization [19]. Interestingly, macrophage depletion completely abrogated MSC-based beneficial effects, while the administration of peritoneal macrophages restored MSC-driven neovascularization in macrophage-depleted animals, confirming that cooperation between MSCs and macrophages was required for successful vascular regeneration [19]. MSCs promote the growth of endothelial cells and induce vascular sprouting in a VEGF-dependent manner [24], while M2 macrophage-derived IL-8 induces the increased expression of VEGFR on endothelial cells, enhancing the pro-angiogenic effects elicited by MSC-sourced VEGF [19]. The generation of smooth muscle cells (SMCs) and pericytes and their recruitment in regenerative vessels are necessary for the development of the mature and functional vasculature [19]. The crosstalk between M2 macrophages and MSCs regulates the differentiation of MSCs in SMCs and pericytes [19,25]. M2 macrophage-sourced TGF-β and prostaglandin F2α are considered as essential paracrine signaling factors for the successful differentiation of MSCs in SMCs, while M2 macrophage-derived PDGF-β is necessary for the optimal differentiation of MSCs in functional pericytes [25].

Crosstalk between MSCs and macrophages is crucially important for the successful engraftment of transplanted MSCs [19]. The survival of exogenously injected MSCs is dependent on the phenotype and function of tissue resident macrophages [19,26,27]. As evidenced in murine models of myocardial infarction and spinal cord injury, anti-inflammatory M2 macrophages, provide a more favorable environment for the engraftment of MSCs than pro-inflammatory M1 macrophages [26,27]. The conversion of pro-inflammatory M1 macrophages to an anti-inflammatory M2 phenotype appears to be critical for the long-term survival of MSCs in healing tissues, suggesting that a mutually beneficial feed-back loop exists between M2 macrophages and MSCs and that the interplay between these cells is crucially important for efficient tissue regeneration [19].

## 4. MSC-Dependent Modulation of NK Cells, Innate Lymphoid Cells and DCs in Injured and Healing Tissues

NK cells are innate immune cells which, due to their potent cytotoxic properties, efficiently eliminate infected and stressed cells at the initial phase of tissue injury and inflammation [28]. However, at the healing phase of tissue repair, under the influence of M2 macrophage-derived immunosuppressive IL-10 and TGF-β, the majority of NK cells acquire anti-inflammatory, regulatory NKreg phenotype and participate in tissue repair and regeneration through the secretion of immunosuppressive IL-10 [28].

MSCs enhance NK cell cytotoxicity at the induction phase of tissue healing, while, at later time points, induce regulatory phenotype or senescence in inflammatory NK cells [29]. At early stage of tissue injury, upon activation of TLR-3, TLR-7 and TLR-9 by viral antigens, MSCs obtain pro-inflammatory (MSC1) phenotype and secrete anti-viral cytokines IFN-α and IFN-β that up-regulate the cytotoxic potential of NK cells (Figure 1) [6,29]. On the contrary, during the resolution phase of tissue injury and inflammation, MSCs in a PGE2- and IDO-dependent manner induce the polarization of inflammatory NK cells into IL-10-producing, anti-inflammatory NKregs (Figure 2) [6,22,29]. Additionally, MSCs produce TGF-β and IL-6, which act synergistically to induce senescence of inflammatory NK cells [29]. Importantly, MSC-generated senescent NK cells exert feedback on MSCs [29]. Senescent NK cells induce a highly significant increase in *VEGF* gene expression in MSCs which, in turn, in VEGF-dependent manner, promotes endothelial cell proliferation and improve vascular regeneration in healing tissues [29].

Innate lymphoid cells (ILCs), a recently discovered heterogeneous group of hematopoietic cells of the innate immune system, are abundant at the mucosal barriers, where they serve as the first responders to tissue injury [30]. GATA-3-expressing type 2 ILCs (ILC2) and RORγtT-expressing type 3 ILCs (ILC3) respond rapidly to alarmins (IL-25, IL-33) released from injured epithelial cells and participate in tissue repair at the barrier surfaces of the skin, airways, and intestine [30].

ILC2 produces Amphiregulin (AREG), a protein that promotes repair of injured lung epithelial cells [30,31,32]. By producing PDGF and IL-7, MSCs induce differentiation of common lymphoid progenitors (CLPs) into AREG-expressing ILC2 cells [33]. MSC-primed ILC2 cells in AREG-dependent manner maintain the integrity of epithelial barrier in the lungs and enhance repair and regeneration of injured lung epithelial cells [33].

RORγt+ ILC3 cells produce IL-22, which protects the integrity of the epithelial cell barrier in the lungs and the gut [30]. MSCs in juxtracrine manner (through the activation of aryl hydrocarbon receptor (AhR)) and in paracrine manner (through the secretion of IL-7) induce the proliferation and activation of AhR and IL-7R-expressing ILC3 [34]. MSC-primed ILC3 showed increased capacity for IL-22 secretion [34]. IL-22, derived from MSC-activated ILC3, increases the synthesis of anti-apoptotic proteins (Bcl-2, Bcl-xL) and proteins that regulate cell cycle (c-Myc, cyclin D1, CDK4) in epithelial cells [30,34]. Accordingly, MSC-primed ILC3, in an IL-22-dependent manner, contribute to wound healing and tissue homeostasis in lungs and intestines by enhancing the viability and proliferation of epithelial cells (Figure 2) [34].

At the healing phase of tissue repair, MSC-derived immunomodulatory factors (IL-6, PGE2, IL-10 and galectin-3), induce generation of tolerogenic phenotype in DCs [6,22]. Tolerogenic DCs are characterized by the reduced expression of co-stimulatory molecules (CD80, CD86 and CD40), down-regulated production of inflammatory cytokines (IL-1β, IL-12, and TNF-α) and increased expression of PDL-1 and PDL-2 [1,3]. Importantly, tolerogenic DCs produce anti-inflammatory cytokines IL-10 and IL-35 and, in an IDO-dependent manner, induce the differentiation of naïve CD4+T cells in immunosuppressive Tregs, which, importantly, contribute to tissue repair and regeneration (Figure 2) [1,3].

## 5. An Interplay between MSCs and Tregs in Tissue Repair and Regeneration

Cells of adaptive immunity, particularly Tregs, also participate in tissue healing [1,3]. CD3^+^CD4^+^CD127^low^CD25^high^Foxp3^+^ Tregs, both thymus-derived (tTregs) and peripherally derived (pTregs), mediate tissue repair by dampening inflammation by modulating the phenotype and function of N1 neutrophils, M1 macrophages, cytotoxic NK cells and pro-inflammatory DCs [35,36]. Tregs, in TGF-β- and IL-10-dependent manners, induce the apoptosis of N1 neutrophils [35]. Additionally, Treg-sourced immunosuppressive IL-10, IL-35 and TGF-β modulate neutrophil and macrophage phenotype and function by promoting their polarization in IL-10, TGF-β, IDO-producing N2 and M2 anti-inflammatory cells [36]. In contact-dependent manner, through the expression of CTLA-4 and LAG-3, Tregs induce generation of tolerogenic phenotype in DCs which, in turn, in IDO-dependent manner, promote expansion of Tregs and create “positive healing loop” in injured tissues [3,35]. In addition to their immunosuppressive properties, Tregs mediate tissue repair by synthesizing “pro-repair” molecules, such as AREG and keratinocyte growth factor (KGF) that directly promote tissue regeneration [31,35,36]. KGF secreted by activated Tregs promotes alveolar epithelial repair, while Treg-derived AREG, an epidermal growth factor receptor (EGFR) ligand, induces mitogenic and cell differentiation signals, enabling reparation of injured muscles, lungs and colons by promoting differentiation of tissue resident stem cells and progenitor cells [31,35,36,37]. Additionally, Tregs may promote tissue regeneration by inducing the proliferation of endothelial and parenchymal cells [38,39]. Treg-sourced AREG, CCL24 and growth arrest specific 6 (GAS6) regulate neonatal heart regeneration by promoting the proliferation of neonatal cardiomyocytes [38] while Treg-derived AREG and IL-10 induce the proliferation of endothelial cells and are mainly responsible for Treg-mediated revascularization and the regeneration of ischemic tissues in diabetic patients [39].

Several lines of evidence demonstrated that efficient tissue repair and regeneration is dependent on the crosstalk between MSCs and Tregs [5,6,22,40,41]. MSCs, in an IDO-dependent manner, induce the degradation of tryptophan (TRP) and generation of immunosuppressive kynurenine (KYN) [22]. KYN promotes expression of Treg lineage-defining transcription factor (forkhead box P3-FoxP3) in CD4+T cells enabling the generation of immunosuppressive CD4+FoxP3+Tregs [22,40,41]. The IDO-mediated degradation of TRP yields a series of KYN catabolites that act as ligands for AhR [22]. The binding of KYN catabolites to AhR and induces conformational changes of AhR that promote its nuclear translocation [6,22]. In the nucleus, AhR induces the enhanced transcription of target genes, including FoxP3 [22]. Accordingly, MSC-sourced IDO, through the activation of KYN/AhR axis results in increased generation of FoxP3+Tregs contributing to the creation of immunosuppressive microenvironment, which will enable efficient tissue healing [22,40,41].

Additionally, during the resolution of tissue injury, MSC-derived IDO prevents the trans-differentiation of IL-10 and IL-35-producing immunosuppressive Tregs in IL-17-producing inflammatory Th17 cells [22]. During initial TCR-mediated activation of resting Tregs, signals via the protein kinase B (PKB/Akt) and mammalian target of rapamycin (mTOR) pathways destabilize the immunoregulatory phenotype of Tregs and cause their reprogramming into a pro-inflammatory helper-like phenotype (“ex-Tregs”), characterized by the enhanced production of inflammatory cytokine IL-17 [22]. Low levels of TRP in the local microenvironment activate stress-response pathways, including the activation of control nonderepressible 2 (GCN2) kinase, which suppresses Akt/mTOR2 signaling. [6,22]. MSC-sourced IDO induces low TRP levels, enabling the activation of GCN2 kinase, which inhibits Akt/mTORC2 signaling in Tregs, preventing their conversion in inflammatory IL-17-producing Th17 cells [22,40,41]. Additionally, MSC-derived IDO and TGF-β may act synergistically to induce conversion of inflammatory Th17 cell in immunosuppressive Tregs [6,22]. Since Th17 cells activate pro-fibrogenic hepatic stellate cells, the IDO-dependent suppression of liver Th17 cells significantly contribute to the attenuation of fibrosis in MSC-treated livers [22,42].

In addition to IDO, MSC-sourced IL-6, PGE2, NO, TGF-β and IL-10 are also responsible for MSC-dependent expansion of Tregs in healing tissues (Figure 2) [22,40,41]. MSCs-sourced NO and PGE2 significantly increase expression of CD62L and CCR7 in Tregs enabling their increased migration into injured tissues [22]. Additionally, MSCs, in IL-6, TGF-β and IL-10-dependent manner induce generation of M2 macrophages which, in turn, in IL-10 and CCL18-dependent manner, recruit Tregs in inflamed tissues, contributing to the creation of immunosuppressive and “pro-healing” microenvironment in injured tissues [3,40,41].

Tregs enhance survival and engraftment of MSCs in ischemic tissues, indicating that the crosstalk between MSCs and Tregs is bidirectional process that enables efficient tissue repair [43]. Tregs may improve pro-angionic properties of MSCs by increasing their capacity for VEGF production [40,41,43]. As recently reported by Zhou and colleagues [43], a significantly increased number of newly generated endothelial cells, associated with improved myocardial function, were noticed in ischemic hearts of Yorkshire pigs which received both Tregs and MSCs compared to experimental animals that were transplanted with MSCs only [43], suggesting that Tregs efficiently improved the pro-angiogenic properties and therapeutic potential of MSCs.

## 6. Concluding Remarks and Future Perspectives

MSCs efficiently enhanced tissue repair and regeneration by modulating the phenotype and function of immune cells (Table 1) [5,13,19,29,40,41]. Immediately after tissue injury, DAMPs and/or PAMPs-primed MSCs obtain pro-inflammatory phenotype and, through the secretion of inflammatory cytokines, increase the cytotoxicity of NK cells, enhance the capacity for antigen-presentation of DCs and improve the phagocytic properties of neutrophils and macrophages, contributing to the removal of microbial pathogens, necrotic tissue and cellular debris [5,13,19,29,40,41]. During the healing phase of tissue repair, MSCs acquire immunosuppressive phenotype and, through the release of immunomodulatory factors and immunoregulatory miRNAs, induce the generation of anti-inflammatory, regulatory and tolerogenic phenotype in neutrophils, macrophages, T cells and DCs, [5,13,19,29,40,41]. Additionally, MSCs produce various pro-angiogenic factors and promote neo-vascularization in healing tissues, contributing to their enhanced repair and regeneration [44].

Despite these promising results, it should be noted that several safety issues raised serious concerns about the clinical use of MSCs [45]. Encapsulated structures, that contained calcifications and ossifications, were observed in the MSC-treated tissues, suggesting that transplanted MSCs may differentiate in unwanted cells (osteocytes and chrondrocytes) under the influence of local tissue microenvironment [45]. In comparison with the MSCs of healthy donors, MSCs derived from patients with inflammatory or metabolic diseases showed significantly impaired proliferation and differentiation potential [46]. These findings should be taken into account whenever MSCs are considered for autologous transplantation in patients with chronic inflammatory and metabolic diseases [46]. Although the transplantation of allogeneic MSCs may circumvent these problems, the risk of possible immunological response against allogeneic MSCs exists [6]. MSCs lack the expression of major histocompatibility complex (MHC) class II and co-stimulatory molecules, but express MHC class I molecules and, therefore, may elicit strong allogeneic immune responses in MHC-class I-mismatched recipients, which may aggravate on-going tissue injury [6].

Importantly, the side effects related to the clinical application of MSCs were not observed in animals and patients that were treated with MSC-derived exosomes (MSC-Exos) [45,47]. Due to their nano-sized dimension and lipid envelope, MSC-Exos, penetrate through the tissue and easily reach the target cells [47]. Through the direct fusion with the plasma membrane of target cells, MSC-Exos deliver their content directly to the cytosol of target cells, without affecting the function of neighboring cells [47]. MSC-Exos contain all of the same immunoregulatory and pro-angiogenic factors as their parental cells and immunomodulation mediated by MSC-Exos was either similar or even better than immunomodulation accomplished by MSCs [47,48]. Accordingly, MSC-Exos represent potentially new immunomodulatory cell-free therapeutic agents in regenerative medicine, the efficacy of which should be explored in up-coming clinical trials.

## Figures and Tables

**Table 1:** The impact of mesenchymal stem cell-derived factors on phenotype and function of immune cells during tissue repair and regeneration.

MSC-Derived Factor(s)	Target Cells	Altered Function of Immune Cells	Immunomodulatory and/ or Regenerative Effects	Ref.
Initial phase of tissue injury				
IL-8; MIF	neutrophils	enhanced phagocytic ability	more efficient removal of necrotic tissue and cellular debris	[12,13]
CCL2; CCL3; CXCL2; CCL12	monocytes	increased chemotactic ability	better recruitment of inflammatory monocytes into the injured tissues	[19,20]
IFN-α; IFN-β	NK cells	increased cytotoxicity	more efficient removal of infected cells	[6,29]
Healing phase of tissue repair				
TSG-6	neutrophils	reduced ROS production; increased IL-10 secretion	creation of immunosuppressive microenvironment	[14]
TGF-β and IL-10	macrophages	generation of anti-inflammatory (M2) phenotype	creation of immunosuppressive microenvironment	[6,7]
TSG-6	macrophages	alleviated secretion of NO, TNF-α and IL-1β	attenuation of on-going inflammation	[21,22]
IL-6 and PGE2	macrophages	increased IL-10 secretion	creation of immunosuppressive microenvironment	[21]
IL-1Ra	macrophages	reduced synthesis of matrix degrading enzymes and inflammatory chemokines	inhibition of IL-1β-driven inflammation	[22,23]
VEGF	macrophages	increased production of IL-8	better neo-vascularization	[25]
PGE2; IDO	NK cells	generation of NKregs	creation of immunosuppressive microenvironment	[22,29]
TGF-β and IL-6	NK cells	induction of senescence	attenuation of on-going inflammation	[29]
PDGF and IL-7	ILC2	generation of AREG-expressing ILC2 cells	enhanced repair of injured epithelial cells	[33]
IL-7	ILC3	increased proliferation and activation of IL-22-producing ILC3	enhanced viability and proliferation of epithelial cells; improved wound healing	[34]
IL-6, PGE2, IL-10; galectin-3	DCs	generation of tolerogenic phenotype	attenuation of on-going inflammation	[6,22]
IDO	Tregs	generation and expansion of Tregs	creation of immunosuppressive microenvironment	[40,41,42]
NO and PGE2	Tregs	increased chemotactic ability	better recruitment of immunosuppressive Tregs into the injured tissues	[22,40,41]

Abbreviations: mesenchymal stem cells (MSCs), interleukin-8 (IL-8), macrophage migration inhibitory factor (MIF), C–C motif chemokine ligand (CCL), C-X-C motif chemokine ligand (CXC), natural killer (NK) cells, interferon alpha (IFN-α), interferon beta (IFN-β), reactive oxygen species (ROS), tumor necrosis factor alpha stimulated gene/protein 6 (TSG-6), interleukin 1 beta (IL-1β), interleukin-6 (IL-6), prostaglandin E2 (PGE2), interleukin-10 (IL-10), interleukin 1 receptor antagonist (IL-1Ra), vascular endothelial growth factor (VEGF), Amphiregulin (AREG), innate lymphoid cells (ILC), indolamine 2,3-dioxygenase (IDO), T regulatory cells (Tregs), NK regulatory cells (NKregs), Platelet-derived growth factor (PDGF), interleukin-7 (IL-7), transforming growth factor beta (TGF-β), innate lymphoid cells type 2 (ILC2); innate lymphoid cells type 3 (ILC3), nitric oxide (NO); dendritic cells (DCs).

## Data Availability

Not applicable.
